# The Pan-Plastome of Walnuts (*Juglans regia* L.) from Xinjiang Reveals New Insights into Their Genetic Diversity

**DOI:** 10.3390/ijms27156935

**Published:** 2026-08-02

**Authors:** Lulu Zhang, Fangdong Geng, Huijuan Zhou, Baoqing Wang, Peng Zhao, Qingguo Ma

**Affiliations:** 1 Research Institute of Forestry, Chinese Academy of Forestry, Beijing 100091, China; 2 Key Laboratory of State Forestry and Grassland Administration on Desert Oasis Ecosystem Protection and Restoration, Xinjiang Key Laboratory of Fruit Tree Species Breeding and Cultivation, Xinjiang Key Laboratory of Forestry and Grassland Sand Control and Desert Industry, Xinjiang Academy of Forestry, Urumqi 830000, China; 3 Key Laboratory of Resource Biology and Biotechnology in Western China, Ministry of Education, College of Life Sciences, Northwest University, Xi’an 710069, China; 4 College of Environment and Life Sciences, Weinan Normal University, Weinan 714000, China; 5 Xi’an Botanical Garden of Shaanxi Province, Institute of Botany of Shaanxi Province, Shaanxi Academy of Sciences, Xi’an 710061, China

**Keywords:** *Juglans regia* L., chloroplast genomes, comparative analysis, phylogenetic evolution

## Abstract

Persian walnut (*Juglans regia* L.) is an economically important tree species in the Juglandaceae family. Comparative analysis of its chloroplast genome sequences is of great significance for species identification and evolutionary research. In this study, high-throughput sequencing was performed using the Illumina HiSeq platform, followed by de novo assembly of the chloroplast genome, and then analyses of its repetitive sequences, codon usage bias, nucleotide polymorphism and population structure. The results showed that the chloroplast genome size of walnut ranged from 160,311 bp to 160,367 bp with a GC content of 36.11%. It exhibited a typical quadripartite circular structure, and a total of 78 distinct protein-coding genes (PCGs), 30 tRNA genes and four rRNA genes were annotated. A total of 16 distinct SSR motifs were identified, among which A/T motifs were the most abundant; 31–40 bp long tandem repeats were the most abundant in all samples, which increased genomic variability. Codon usage bias analysis revealed that all walnut samples preferentially use the codon UUA (Leu), and most codons end with A/U. Nucleotide sequence alignment identified 129 polymorphic sites. Single nucleotide variants (SNVs) were the dominant variation type, followed by InDels. Furthermore, the phylogenetic tree, principal component analysis (PCA), haplotype network and population structure classified the 41 walnut samples into three genetic clusters (C1, C2 and C3). This study elucidated the conserved characteristics and evolutionary variations in walnut chloroplast genomes, providing theoretical support for its phylogenetic research and germplasm resource utilization.

## 1. Introduction

Persian walnut (*Juglans regia* L.), also known as common walnut [1], is a deciduous tree of the *Juglans* genus in Juglandaceae, and China has the world’s largest walnut production [2,3]. *Juglans* is an economically important species with a long cultivation history, abundant species diversity and wide distribution range [4]. Walnut kernels are rich in high-quality fat, proteins, minerals and vitamins that contribute to human health [5]. Research has shown that the level of unsaturated fatty acids in walnut oil is as high as 90%, which has various functions including antioxidation, hypoglycemia, liver protection and promotion of fatty acid metabolism, and can be used as components of drugs in healthcare [6]. Meanwhile, the protein content is 15%~20%, and the absorbable protein content reaches as high as 96%, which has roles in antihypertension and anticancer [7]. In addition, it contains minerals and vitamins, such as calcium and vitamin E [8]. Walnut is widely valued for its high kernel oil yield and exceptional quality timber, and possesses nutritional, medicinal and economic values [9].

China is one of the original distribution centers of walnut with abundant walnut resources. The native *Juglans* species in China include *Juglans regia*, *Juglans sigillata*, *Juglans mandshurica*, *Juglans hopeiensis* and *Juglans cathayensis*, with *Juglans regia* and *Juglans sigillata* having the widest cultivation area [10]. China is also regarded as an important center of walnut diversity, providing rich germplasm resources for the cultivation and breeding of walnut [11]. Xinjiang is an important walnut producing area in China [12], with unique ecology and soil conditions. It has excellent walnut cultivars, including ‘Wen 185’, ‘Xinxin 2’, ‘Zha 343’ and ‘Xinfeng’, with early-bearing, thin-shell and disease-resistant characteristics [13,14]. However, studies on the genetic diversity and evolutionary history of Xinjiang walnuts remain limited. In previous studies, researchers used the whole genome data of ancient walnut samples from Xinjiang, revealing that walnuts originated from and were domesticated in Central Asia and spread eastward before the Tang Dynasty; Xinjiang served as a crucial transmission hub into China [15].

Plants possess three relatively independent genomes: nuclear genomes, mitochondrial genomes (mtDNA), and chloroplast genomes (cpDNA); the chloroplast and mitochondrial genomes are collectively referred to as organellar genomes [16]. The nuclear genomes contain majority of the genetic information, but organellar genomes still play crucial roles in eukaryotes [17]. Plant organellar genomes are characterized by high conservation and cytoplasmic inheritance, and can provide strong support for studies on species origin and evolution, phylogeny and molecular ecology [18]. Compared with the mitochondrial genome, the plant chloroplast genome is essential for energy metabolism and environmental adaptation; it can drive the evolution and diversity of plants, and has broader applications in molecular evolution and phylogenetic analysis [19]. In recent years, researchers have investigated the chloroplast genomes of terrestrial plants, including *Rhododendron*, *Pelargonium* and *Allium prattii* [20,21,22]. However, relatively few studies have been conducted on the chloroplast genome of Xinjiang walnuts.

Plant chloroplast genomes usually have high structural conservation. The typical chloroplast genome of angiosperms is a circular double-stranded DNA molecule that encodes a variety of genes, including protein-coding genes, tRNA genes and rRNA genes. The majority of chloroplast genomes present a typically circular quadripartite structure, consisting of small (SSC) and large (LSC) single-copy regions, which are separated by a pair of inverted repeat sequences (IRa and IRb) [23]. Studying codon usage bias can enhance the expression level of exogenous genes in chloroplasts, which is of great significance for the genetic improvement of the species [24]. Chloroplast genomes generally exhibit a strong AT mutational bias, and such sequence-dependent mutational features profoundly shape the distribution of synonymous codons, providing a fundamental evolutionary context for dissecting the formation of codon usage bias [25]. Additionally, the chloroplast genome contains a large number of repeated sequences, which are the primary cause of duplication, deletion and rearrangement events in chloroplast genomes [26]. Although chloroplast genomes are highly conserved, genomic structural variations have been detected in many plant species [27]. For example, insertion/deletions (InDels) induced by tandem repeats, inversions, translocations and genomic rearrangements can be used as molecular genetic markers for the evaluation of genetic diversity, and they have significant effects on the genetic breeding, germplasm identification and phylogenetic analysis of species [28]. Single nucleotide variants (SNVs) are efficient molecular markers that can facilitate the screening of superior genes and shorten the breeding cycle [29]. Phylogenetic analysis of walnut populations can clarify differentiation relationships and reveal the evolutionary rules. Currently, due to the wide distribution of walnut germplasm resources and the lack of a systematic comparison, problems remain in the research on evolutionary relationships and population genetics.

In this study, the chloroplast genomes of 41 samples of *J. regia* were assembled and assessed. The research objectives include the following: (1) analyzing its genome characteristics, gene composition, codon usage bias and repetitive sequences; (2) revealing the evolutionary relationships through comparative analysis with related species and phylogenetic analysis; (3) evaluating the genetic diversity structure of walnut populations in Xinjiang. These studies will further deepen the understanding of the structure and function of the chloroplast genome, and provide important theoretical support for the genetic improvement and resource utilization of walnuts.

## 2. Results

### 2.1. Pan-Plastome Structure and Organization

**Figure 1:**
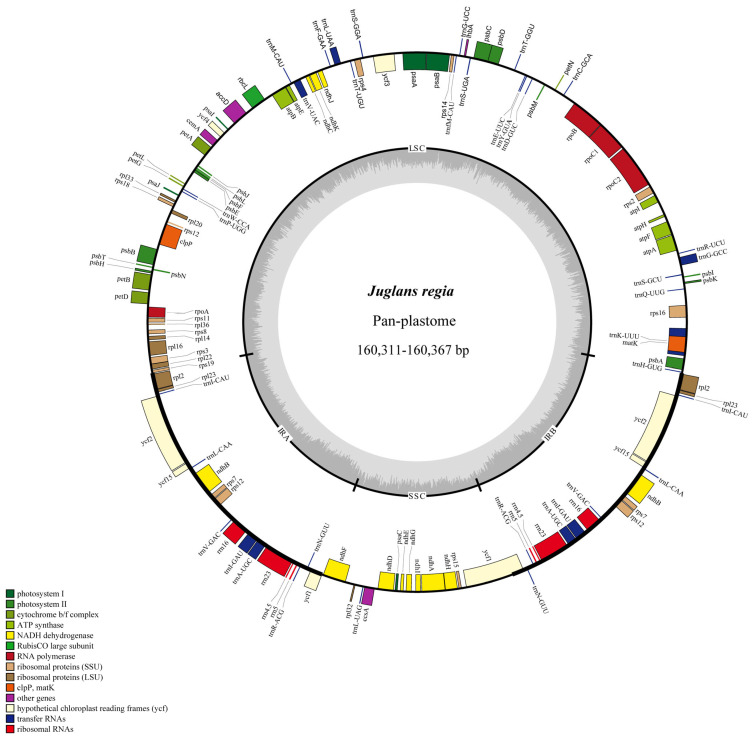
The pan-plastome of walnut germplasms from Xinjiang. The inner genes of the outer circle are transcribed counterclockwise, while the outer genes are transcribed clockwise; genes with introns are marked with an asterisk (*clpP*, *ycf3* and *rps12* contain two introns, and all others contain one). Dark gray bars on the inner circle indicate GC content.

In this study, 41 plastome samples were successfully assembled using resequencing data. All assembled plastomes possessed a typical quadripartite structure, including an LSC, SSC, and a pair of inverted repeat regions (IRA and IRB). The full length of the plastomes of all the samples ranged from 160,311 bp to 160,367 bp, with an average length of 160,359 bp (the median length was 160,367 bp, Figure 1). The IRs across samples were 26,033 bp in length. The GC content of the complete plastomes ranged from 36.11 to 36.12% (the mean and median were both 36.11%).

A total of 78 unique protein-coding genes (PCGs), 30 tRNA genes, and four rRNA genes were annotated and characterized in the plastomes of walnut germplasms from Xinjiang (Table 1, Figure 1). The genes identified were distributed in the genome as follows: 83 genes in the LSC region, which consisted of 61 PCGs and 22 tRNAs; 12 genes in the SSC region, which consisted of 11 PCGs and one tRNA; and 17 genes in the IR regions, which included six PCGs, four rRNA genes, and seven tRNAs. All genes in the IR regions had two copies (one in each IR) and were counted only once. The *trnK-UUU*, *rps16*, *trnG-GCC*, *atpF*, *rpoC1*, *trnL-UAA*, *trnV-UAC*, *petB*, *petD* and *rpl16* genes were located in the LSC region containing one intron each, while *clpP* and *ycf3* each contained two introns. In the SSC region, ndhA* contained one intron. In the IR regions, rpl2*, ndhB*, trnI-GAU*, and trnA-UGC* each contained one intron. The *ycf1* gene spanned the junctions of the IRB/SSC and SSC/IRA. *Rps12* is a trans-splicing gene the exon of which is distributed in the LSC, IRA, and IRB regions. Exon 1 is a single copy in the LSC, and tandem exon 2 and 3 are duplicated in IRAand IRB. Distant coding segments produce separate precursor RNAs, which are ligated via trans-splicing into functional full-length mRNA.

### 2.2. Pan-Plastome Repetitive Sequence

**Figure 2:**
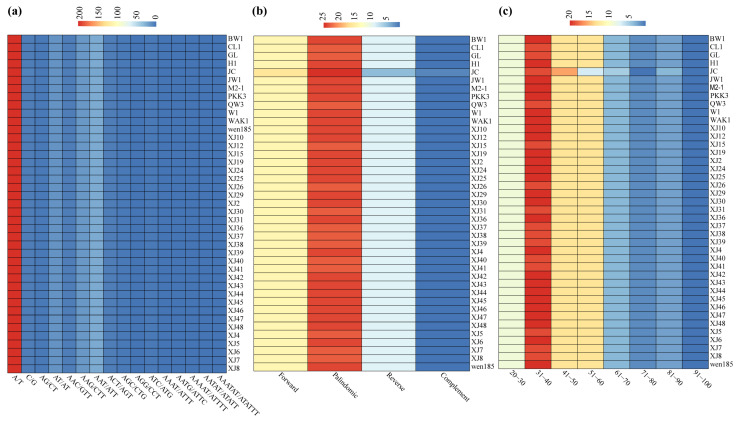
Abundance of repeat sequences annotated in walnut. (**a**) A total of 16 SSR motifs were detected; (**b**) interspersed repeat sequences (C, complement; F, forward; P, palindromic; R, reverse); (**c**) long tandem repeat sequences with lengths ranging from 20 bp to 100 bp in 10 bp increments.

The SSR search revealed 16 different SSR motifs, including two homopolymer SSRs, two dinucleotide SSRs, seven trinucleotide SSRs, two tetranucleotide SSRs, two pentanucleotide SSRs, and one hexanucleotide SSR, among which variants of the A/T type motifs were the most abundant. The AAT/ATT motif was second in abundance to A/T. Among the pentanucleotide SSRs, AATAT/ATATT was identified in only one sample (JC), in which there was only one (Figure 2a). REPuter detected four types in the plastomes of all samples: forward (F), palindromic (P), reverse (R) and complementary (C) repeats, with P repeats being the most abundant and C repeats being the least abundant (order of abundance: P > F > R > C; Figure 2b). TRF was used to identify long tandem repeats of different lengths (20–100 bp). The 31–40 bp repeat category was the most abundant in all samples, with an average of 20 repeats. Samples BW1, H1, JW1, M2-1, PKK3, W1, WAK1, XJ10, XJ12, XJ19, XJ2, XJ24, XJ25, XJ29, XJ30, XJ36, XJ4, XJ42, XJ43, XJ44, XJ45, XJ46, XJ47, XJ48, XJ6 and Wen185 had the highest abundance, with 20 such repeats. The 41–50 bp averaged 13 repeats per plastome (Figure 2c). Among them, sample JC had the greatest number of repeats in this size category (with 16 repeats each). At 51–60 bp, JC had the least amount, with seven repeats each. In the 71–80 bp category, JC had the highest amount, with five such repeats. At 81–90 bp, JC had the highest amount, with four such repeats. At 91–100 bp, all samples had only one repeat.

### 2.3. Codon Preference Analysis

**Figure 3:**
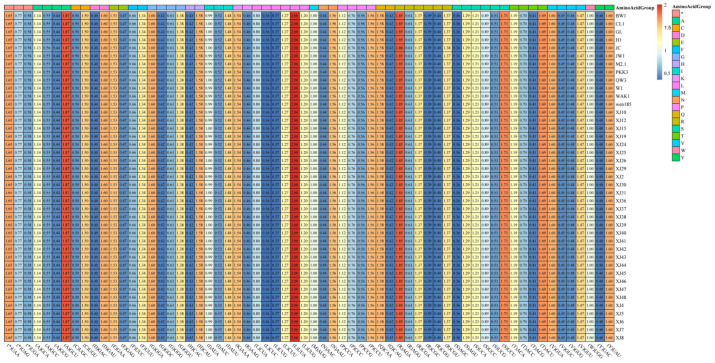
Relative synonymous codon usage (RSCU) in the chloroplast genome of walnut. * Three stop codons (UAA, UAG, UGA).

**Figure 4:**
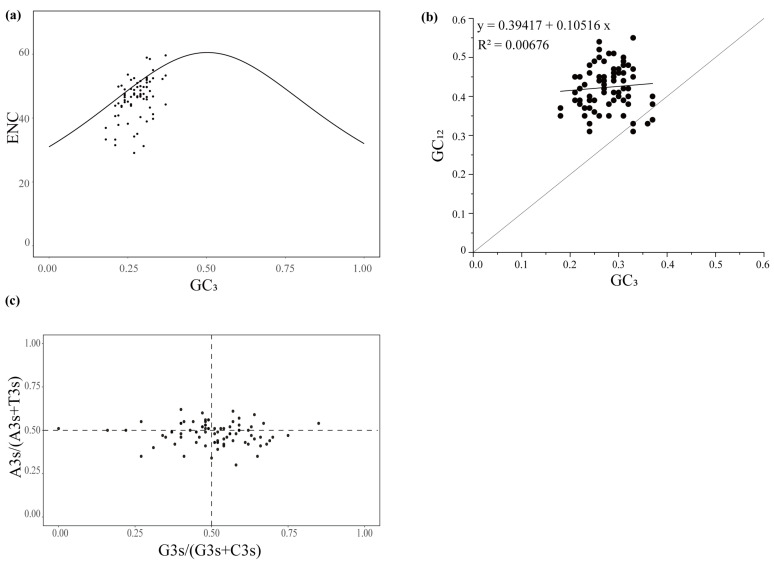
Plots of the causes of codon preference in the chloroplast genome. (**a**) ENC-plot analysis. The curve in the plot represents the expected curve of gene positions when codon usage is determined by GC_3_ composition, (**b**) neutrality analysis, where the diagonal line represents the neutral expectation (Y = X), (**c**) PR2-bias plot. The two dotted lines are 0.5 baselines. Black dots represent distinct genes.

Codon preference is commonly quantified by the relative synonymous codon usage (RSCU), with values ranging from 0 to 2. A codon with relative synonymous codon usage (RSCU) > 1 is considered to be frequently used by the amino acid. Analysis of the CDS in the chloroplast genome of all walnut samples revealed variability among different lineages in codon usage. In high frequency codons (RSCU > 1), the third codon position was mostly A/U. Among low frequency codons (RSCU < 1), the third codon position was mostly G/C. This is a common characteristic of codon preference in organelle genomes of terrestrial plants. All walnut samples preferentially used UUA in coding Leucine, which had the highest RSCU value in the chloroplast CDS (RSCU = 2). Analyses of the plastome CDSs of all tested samples revealed variability among different lineages in codon usage. For instance, the most frequently used synonymous codons were GCU (Ala, RSCU = 1.87), and AGA (Arg, RSCU = 1.85) in all samples. The least frequently used synonymous codons were CUC (Leu, RSCU = 0.36), CUG (Leu, RSCU = 0.37), and AGC (Ser, RSCU = 0.36). The AUG (Met) had an RSCU of =1 (Figure 3). To explore the correlation between nucleotide composition and codon usage bias, ENC-GC_3_ correlation analysis was performed. The results revealed that the ENC values of most genes fell below the theoretical standard ENC curve, whereas only a small number of genes conformed to the theoretical expectation (Figure 4a). Neutrality plot analysis showed that GC_12_ values ranged from 0.31 to 0.55, while GC_3_ values varied between 0.18 and 0.37, with most points distributed above the diagonal line. The slope of the regression line (regression coefficient) was 0.10516, indicating that mutational pressure accounted for 10.52% of the codon usage variation, whereas natural selection contributed 89.48%. Furthermore, regression analysis between GC_12_ and GC_3_ revealed an extremely weak correlation between the two parameters (R^2^ = 0.00676). Collectively, mutational pressure and natural selection jointly modulate codon usage bias (Figure 4b). PR2 plot analysis revealed that most genes were distributed in the bottom-right quadrant, indicating that T occurred more frequently than A, while G was utilized more often than C at the third codon position. Such nucleotide imbalance demonstrates that mutational pressure and natural selection jointly shape the bias in codon usage (Figure 4c).

### 2.4. Pan-Plastome Polymorphisms

To resolve differences in all plastomes at the nucleotide level, 41 newly assembled walnut complete plastomes were used for the following analyses. A total of 129 nucleotide polymorphism sites were identified in all walnut samples, including the coding DNA sequences (CDSs), exons, introns and IGSs (gene intervals). The most diverse variation type was SNVs, with 72 (55.81%), followed by InDels, with 54 (41.86%), and the least was substitutions, with three (2.33%). Based on the results, polymorphism was further analyzed: CDSs contained 20 SNVs, zero InDels and zero substitutions; introns contained eight SNVs, 12 InDels and zero substitutions; and IGSs contained 44 SNVs, 42 InDels and three substitutions. The results show that IGSs contained the most number of polymorphisms (89, 68.99%), followed by CDSs and introns (20, 15.50%).

**Figure 5:**
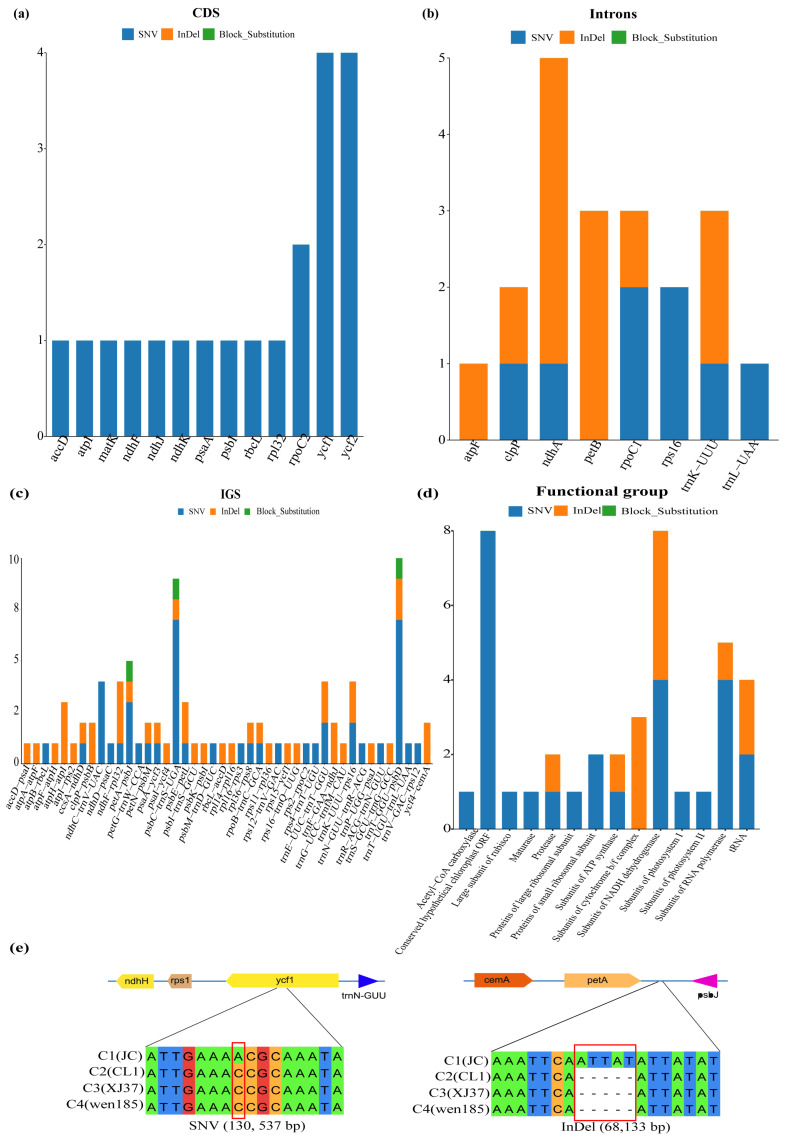
Variant location categorized by genic position. (**a**) CDS, (**b**) introns, (**c**) IGS, (**d**) functional grouping, (**e**) left: SNV in the *ycf1*. right: InDel in the *petA-psbJ*. Red boxes indicate variable sites. Different colors correspond to four nucleotides: green for A, blue for T, red for G, and orange for C.

We analyzed the polymorphisms in each gene in the pan-plastome with the *ycf1* gene, which lies in the boundary regions between IRB/SSC and SSC/IRA. In CDS, *ycf1* and *ycf2* have the highest polymorphism, with no InDels (Figure 5a). Among introns, *ndhA* contained the most polymorphic sites, *atpF* and *trnL-UAA* contained the fewest polymorphisms, while the *ndhA* gene was located in the SSC (Figure 5b). In the IGS regions, the site with the highest concentration of polymorphisms was *trnT-GGU-psbD*, followed by *psbC-trnS-UGA* and *petA-psbJ* (Figure 5c). Among the functional groups, NADH dehydrogenase and conserved hypothetical chloroplast ORF contained the most polymorphisms (Figure 5d). A single nucleotide variant (SNV) specific to the C1 lineage was identified within the *ycf1* gene, while an insertion variant (InDel) was detected in the *petA-psbJ* intergenic region of the C1 lineage. These two polymorphic sites can serve as diagnostic markers to distinguish the C1 lineage from the other lineages (Figure 5e). These results indicate that the SSC is the region evolving most rapidly within the plastomes.

### 2.5. Population Structure and Genetic Diversity

**Figure 6:**
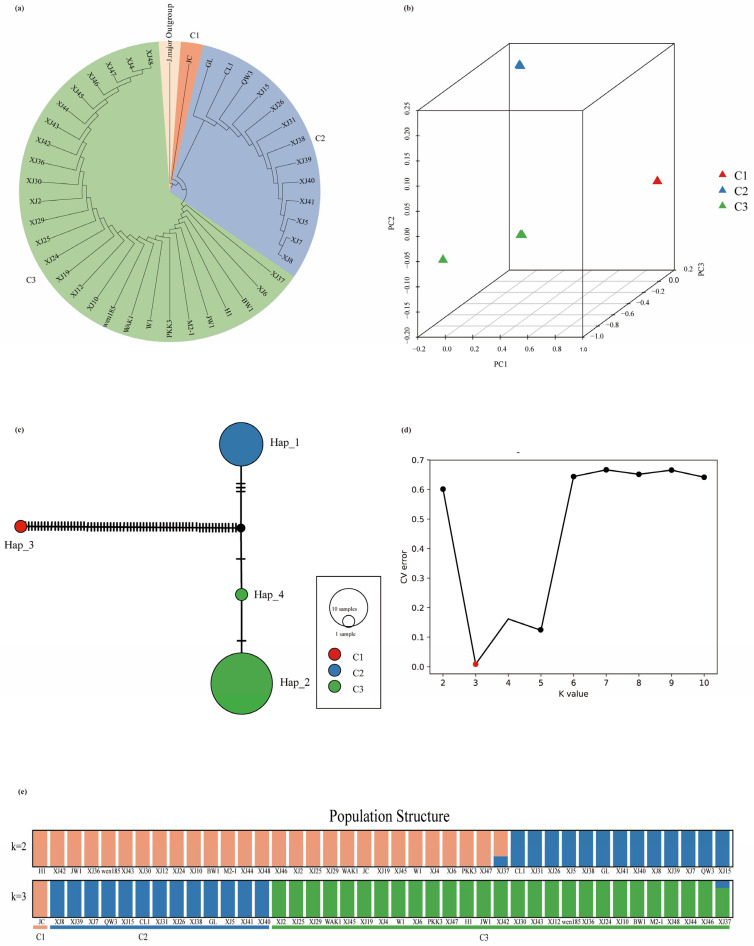
Population structure of walnut samples from Xinjiang. (**a**) NJ tree of 41 samples; (**b**) PCA; (**c**) haplotype network of cpDNA, different colors represent different genetic clusters, vertical tick marks on the branches represent mutational steps. (**d**) CV errors across a range of K values from 2 to 10 with the lowest CV error at K = 3; (**e**) population structure bar plot at K = 2~3.

The NJ tree based on the walnut chloroplast genome shows that C1 lineage was the most recently diverged from outgroups *Juglans major*. The sample JC was assigned to the C1 genetic cluster (Figure 6a). Thirteen samples were assigned to the C2 genetic cluster: GL, CL1, QW3, XJ15, XJ26, XJ31, XJ38, XJ39, XJ40, XJ41, XJ5, XJ7, XJ8. Twenty-seven samples were assigned to the C3 genetic cluster: XJ37, XJ6, BW1, H1, JW1, M2-1, PKK3, W1, WAK1, wen185, XJ10, XJ12, XJ19, XJ24, XJ25, XJ29, XJ2, XJ30, XJ36, XJ42, XJ43, XJ44, XJ45, XJ46, XJ47, XJ4, and XJ48. According to the PCA, the forty-one samples were divided into three genetic clusters, which is consistent with the NJ results (Figure 6b). Based on the haplotype results, the forty-one walnut samples were classified into four haplotypes. Genetic cluster C1 and C2 contained one haplotype each, while C3 had two haplotypes; this was consistent with the NJ and PCA results (Figure 6c). In order to understand lineage relationships among different samples, we used several methods to analyze the walnut population structure and evaluate the genetic diversity among different sample populations. Based on the completed plastomes, we used ADMIXTURE to analyze the population structure. At K = 2, walnut samples were not well distinguished; at K = 3 (the best-supported partition), walnut samples were better resolved to be divided into three genetic clusters (Figure 6d). Sample XJ37 was assigned to two genetic clusters, C2 and C3, while other samples were without cross-cluster assignment (Figure 6e). In addition, the results of the NJ, PCA and haplotype networks all supported the ADMIXTURE findings. Walnut samples were divided into three genetic clusters.

## 3. Discussion

### 3.1. Structural Characterization of Juglans regia Chloroplast Genome

Chloroplasts are the metabolic center of green plants, they transform solar energy into carbohydrates to sustain the life activities of organisms through photosynthesis [30]. Chloroplasts are semi-autonomous organelles that contain their own genetic DNA and have a small genome size. With the rapid development in high-throughput sequencing techniques and assembly technologies, high-quality organelle genome data have been increasingly published, which has promoted more in-depth development in phylogenomics and population genomics research [31]. In this current study, the average length of the 41 walnut chloroplast genomes was approximately 160,359 bp, with a GC content ranging from 36.11% to 36.12%. All assembled genomes exhibited a typical tetrad circular structure, and their genomic features were consistent with those of *Nicotiana*, *Euonymus*, *Ligustrum* and other species [32,33,34]. There are differences in chloroplast genome size among different species, whereas cpDNA was highly conserved. Previous studies have shown that most angiosperm chloroplast genomes contain approximately 110 unique genes, such as *Meliaceae* [35], and *Cardamine* [36]. Consistent with previous study results, a total of 112 unique genes were annotated in the walnut chloroplast genome, comprising 78 protein-coding genes (PCGs), 30 tRNA genes, and four rRNA genes. Similarly to most angiosperms, the chloroplast genome had variable GC content in different regions; the IR regions had the highest GC content among the four regions, followed by the LSC and SSC regions [37]. The IR region had four rRNA genes. Accordingly, the higher GC content in the IR region is likely associated with the distribution of rRNA genes, and high GC content maintains the structural stability of cpDNA.

### 3.2. Variation and Evolution of Chloroplast Genomes

Chloroplast genomes are generally conserved; nevertheless, accumulating evidence reveals substantial variations at certain levels. In this study, the 41 walnut chloroplast genomes ranged in size from 160,311 bp to 160,367 bp, indicating genome size variation. In the present study, the 129 nucleotide polymorphism sites included coding DNA sequences (CDSs), exons, introns, and intergenic spacers (IGSs), which were mainly concentrated in the IGS regions. The *ycf1* gene was also found to have most variants in other species [38]. Further analysis found that A > C nucleotide variation was detected in the *ycf1* gene, which can distinguish the C1 group from other groups. An InDel was detected in *petA-psbJ*, which can be used as a molecular marker to distinguish the C1 genetic cluster from others. The CDSs only contained single nucleotide variations (SNVs), while the *ycf1* and *ycf2* genes have a higher number of SNVs than other genes. Both SNVs and InDels were present in intron regions, and the *ndhA* gene showed the largest number of InDel polymorphic sites. The intergenic spacers (IGSs) displayed the most abundant genetic variation; *trnT-GGU-psbD*, *psbC-trnS-UGA* and *petA-psbJ* exhibited the greatest nucleotide polymorphism. Among the functional groups, the acetyl-CoA carboxylase and NADH dehydrogenase genes contained the largest number of polymorphic sites. Similarly to the findings for *Hemerocallis citrina*, these nucleotide variations can be used to develop molecular markers for identifying maternal inheritance in walnut [39]. These findings illustrate the important role of highly variable non-coding regions in plant phylogenetic studies, which support the evolutionary conservation of coding regions.

Repeat sequences play an important role in the rearrangement, divergence and evolution of chloroplast genomes [40]. Studies on *Aconitum* have indicated that structural variations are mediated by repeat sequences, with long repeats showing a strong correlation with chloroplast genome rearrangements [41]. Studies have shown that SSRs can reveal genetic differences at the DNA level that are not affected by environmental factors, which can be used as molecular markers, in species evolution, and in cultivar identification [42]. In this study, 16 distinct SSRs were found in the walnut chloroplast genome; A/T motif repeats were the most abundant, which is consistent with the findings of other *Juglans* species [43]. In addition, the long repeat sequences of the species can reflect its evolutionary degree. In all samples of this study, the 31–40 bp tandem repeats were the most abundant, followed by the 41–50 bp repeats. This finding suggests that these repeat sequences may facilitate genome rearrangement. These SSRs and long repeat sequences can be used to study genetic structure diversity and evolution.

The usage of synonymous codons is an important factor affecting gene expression and genome evolution, and it is of great significance for studying the origin and genetic differentiation of species [44]. In this study, 30 codons had an RSCU value greater than 1. Most codons ended with A/U at the third codon position. Leucine (Leu) tended to be encoded by the UUA, which showed the highest RSCU value (RSCU = 2) among all chloroplast CDSs of walnut samples. Alanine (Ala) encoded by the GCU codon ranked second (RSCU = 1.87). These codon usage patterns were consistent with previous findings on base composition characteristics in *Fabaceae* and *Solanum* species, which is in agreement with the general codon usage pattern of plant chloroplast genomes [45,46]. Codon usage bias (CUB) is affected by multiple factors, among which natural selection and mutational pressure constitute the primary determinants [47]. ENC-plot analysis showed that the majority of genes fell below the theoretical ENC curve. Furthermore, neutrality plot analysis revealed that most data points clustered above the diagonal line with a regression slope of 0.10516, demonstrating that codon usage bias (CUB) is comodulated by mutational pressure and natural selection. PR2 parity rule analysis indicated that most genes were distributed in the bottom-right quadrant, where T appeared more frequently than A and G was preferentially utilized over C at the third codon position, further illustrating that mutational pressure and natural selection jointly shaped codon usage bias. The results are consistent with previous studies on *Sapindaceae*, *Manihot esculenta*, and *Actinostemma tenerum* [48,49,50]. The results of the present study support that natural selection plays a moderate role in shaping codon usage bias. Nevertheless, several previous studies have demonstrated that chloroplast genomes of plants exhibit pervasive context-dependent mutation dynamics; the flanking base compositions substantially alter substitution rates and equilibrium base compositions, forming the dominant mutational pressure governing the codon usage patterns of most genes [51]. Future analyses of codon preference should integrate context-dependent mutation models to achieve more accurate evaluations [52].

### 3.3. Genetic Structure and Population Differentiation

All analyses including NJ, ADMIXTURE, PCA and haplotype network methods, yielded consistent clustering patterns, which clustered the 41 samples into three genetic groups. The NJ tree was constructed based on the genetic distances of walnut chloroplast genomes. Neighbor-joining (NJ) cluster analysis was used to explore the evolutionary relationships among the 41 walnut samples. Lineage C1 recently diverged from the outgroup *Juglans major*, and only JC was assigned to the C1 genetic cluster. Lineages C2 and C3 diverged due to geographical isolation; 13 samples were assigned to genetic cluster C2, and 27 samples belonged to cluster C3, indicating that lineage C3 likely has the highest genetic diversity. The PCA supported the genetic structure results, indicating that there is a significant population genetic structure among different samples. The haplotype analysis results showed that the 41 walnut samples were divided into four haplotypes. The C1 and C2 genetic clusters each contained one haplotype, and the C3 genetic cluster contained two haplotypes. Furthermore, to further reveal the genetic structure of the 41 accessions, clustering analysis was performed (K = 2~10). At K = 3 (the optimal partition), the samples were divided into three genetic clusters, except for sample XJ37, which was assigned to two genetic clusters (C2 and C3); all other samples were clearly grouped without cross-classification. These results indicate the presence of gene flow, which is consistent with previous studies on *Juglans nigra* [53]. Walnut samples from the same region belonged to different genetic clusters, suggesting a complex cultivation history of walnuts. Based on the above analytical results, further studies with larger sample sizes are required to obtain large-scale genetic data in the future.

## 4. Materials and Methods

### 4.1. Plant Material and Sequencing

Forty-one samples were collected from Xinjiang (Table S1). The healthy leaves were taken and immediately frozen in liquid nitrogen. After their removal, they were stored at −80 °C until DNA isolation. Genomic DNA was extracted using the CTAB method [54]. The extracted DNA was sequenced using the Illumina HiSeq platform (Illumina, San Diego, CA, USA) to generate raw paired-end reads. Fastp was used to perform quality control on raw reads to obtain high-quality reads [55].

### 4.2. Plastome Assembly and Annotation

Clean data were assembled de novo using SPAdes v3.14.1 [56] with k-mer sizes set to 21, 45, 65, 85 and 105, and graph files were generated accordingly. Subsequently, Bandage v0.8.1 was used for visual analysis to remove redundant contigs and construct circular contigs [57]. The circular sequences were regarded as the complete chloroplast genome sequences of walnut. The assembled complete chloroplast genomes were annotated using PGA v1.3 [58], with MF167463.1 and NC_035966.1 as reference sequences, followed by manual curation and correction of the annotation results. OGDRAW v1.3.1 [59] was employed to visualize the chloroplast genomes and illustrate their genomic structural features.

### 4.3. Codon Usage Bias

CodonW 1.4.2 [60] was used to calculate the relative synonymous codon usage (RSCU) codon usage indices T3s, C3s, A3s, and G3s (the frequency of T/C/A/G usage at the third position), and the effective number of codons (ENC). EMBOSS [61] was employed to calculate the GC contents at three discrete codon positions (GC_1_, GC_2_ and GC_3_), the average GC content of GC_1_ and GC_2_ was computed for statistical quantification (GC_12_). Linear regression analysis was performed to assess the correlation between GC_12_ and GC_3_. The values of ENC and GC_3_ were visualized using the R “ggplot2” v3.4.4, and the standard ENC curve was constructed simultaneously. The PR2 plot was constructed based on GC bias G_3_/(G_3_ + C_3_) and AT bias A_3_/(A_3_ + T_3_) to evaluate the effects of mutational pressure and natural selection pressure.

### 4.4. Repeat Sequences

Simple sequence repeats (SSRs) were identified using MISA v1.0 [62], with the parameter setting of 1–10, 2–5, 3–4, 4–3, 5–3 and 6–3, to detect SSR across the genome. Dispersed repetitive sequences were screened by vmatch v2.3.0 [63], with the minimum length set to 30 bp and a Hamming distance of 3, for the identification of qualified dispersed repeats. Tandem repetitive sequences were detected using TRF (trf409.linux64) [64], with the parameter configuration: 2 7 7 80 10 50 2000 -f -d -m, to accurately locate tandem repeat regions in the chloroplast genome.

### 4.5. Population Structure and Phylogenetic Analysis

MEGA7 [65] was used to construct the neighbor-joining (NJ) phylogenetic tree, with a bootstrap value set to 1000 to evaluate tree reliability and reveal the evolutionary relationships among samples. Principal component analysis (PCA) was performed using GCTA v1.94.0 [66] to explore major genetic variation components and clarify the genetic structure and population differentiation. DnaSP v6 [67] was applied to identify haplotypes based on aligned consensus gene sequences and classify distinct haplotype types. POPART v1.7 [68] was employed to construct a TCS haplotype network, visually illustrating the evolutionary relationships and genetic distances among different haplotypes. Population genetic structure analysis was conducted using ADMIXTURE v1.3.0 [69] to infer genetic grouping and the proportion of ancestral genetic components of each sample.

### 4.6. Haplotype and Genetic Diversity Analyses

Multiple sequence alignment was automatically performed using MAFFT v7.427 [70] to ensure sequence comparability. DnaSP v6 [67] was used to detect variation sites, including single nucleotide variants (SNVs) and insertions/deletions (InDels), and to analyze the genetic diversity of the aligned sequences.

## 5. Conclusions

In this study, the pan-plastome of *J. regia* was successfully assembled using samples collected from Xinjiang, which was 160,359 bp in length with a GC content of 36.11%. It encoded 78 distinct protein-coding genes (PCGs), 30 tRNA genes, and four rRNA genes. We systematically analyzed genomic features including size variation, repetitive sequences, codon usage bias, and nucleotide diversity. The results revealed that, among the 41 walnut samples, the SSC region exhibited the highest variability, and repetitive sequence analysis indicated genetic divergence among these samples. High-frequency codons mostly terminate with A/U at the third codon position. Comprehensive analyses of ENC-plot, neutrality plot and PR2 parity rule revealed that codon usage bias (CUB) in the chloroplast genome of *J. regia* confers preference for A and U. Comparative genomic analysis reveal that the chloroplast genomes of walnut exhibit substantial variations in both size and structure. This study identified 129 nucleotide polymorphic sites, as well as several highly variable regions, such as *ycf1*, *ycf2* and *petA-psbJ*, which can serve as potential molecular markers. Population structure analysis classified the 41 walnut samples into three genetic clusters (C1, C2 and C3). These findings will contribute to an in-depth understanding of the characteristics of organellar genomes in walnut, and provide an important basis for further phylogenetic evolution studies and marker-assisted breeding.

## Figures and Tables

**Table 1:** Annotated genes in walnut germplasms from Xinjiang plastomes.

Gene Category	Functional Group	Gene Name
protein-coding genes	Subunits of photosystem I	*psaA*, *psaB*, *psaC*, *psaI*, *psaJ*
	Subunits of photosystem II	*psbA*, *psbB*, *psbC*, *psbD*, *psbE*, *psbF*, *psbH*, *psbI*, *psbJ*, *psbK*, *psbL*, *psbM*, *psbN*, *psbT*
	Large subunit of ribosome	*rpl14*, rpl16 *, rpl2 * (2), *rpl20*, *rpl22*, *rpl23(2)*, *rpl32*, *rpl33*, *rpl36*
	Small subunit of ribosome	*rps11*, rps12 ** (2), *rps14*, *rps15*, rps16 *, *rps18*, *rps19*, *rps2*, *rps3*, *rps4*, *rps7(2)*, *rps8*
	NADH dehydrogenase	ndhA *, ndhB * (2), *ndhC*, *ndhD*, *ndhE*, *ndhF*, *ndhG*, *ndhH*, *ndhI*, *ndhJ*, *ndhK*
	Cytochrome b/f complex	*petA*, petB *, petD *, *petG*, *petL*, *petN*
	Subunits of ATP synthase	*atpA*, *atpB*, *atpE*, atpF *, *atpH*, *atpI*
	RNA polymerase	*rpoA*, *rpoB*, rpoC1 *, *rpoC2*
	Large subunit of Rubisco	*rbcL*
	Conserved hypothetical chloroplast ORF	*#ycf15(2)*, *lhbA*, *ycf1(2)*, *ycf2(2)*, ycf3 **, *ycf4*
	c-type cytochrome synthesis gene	*c* *csA*
	Envelope membrane protein	*c* *emA*
	Protease	clpP ** ^b^
	Acetyl-CoA carboxylase	*Acc* *D*
	Maturase	*matK*
tRNA genes	Transfer RNA	trnA-UGC * (2), *trnC-GCA*, *trnD-GUC*, *trnE-UUC*, *trnF-GAA*, trnG-GCC *, *trnG-UCC*, *trnH-GUG*, *trnI-CAU(2)*, trnI-GAU * (2), trnK-UUU *, *trnL-CAA(2)*, trnL-UAA *, *trnL-UAG*, *trnM-CAU*, *trnN-GUU(2)*, *trnP-UGG*, *trnQ-UUG*, *trnR-ACG(2)*, *trnR-UCU*, *trnS-GCU*, *trnS-GGA*, *trnS-UGA*, *trnT-GGU*, *trnT-UGU*, *trnV-GAC(2)*, trnV-UAC *, *trnW-CCA*, *trnY-GUA*, *trnfM-CAU*
rRNA genes	Ribosomal RNA	*4.5S rRNA (2)*, *5S rRNA (2)*, *16S rRNA (2)*, *23S rRNA (2)*

* Gene with one intron. ** Gene with two introns. # Gene: Pseudo gene. ^b^ Gene with two introns. (2) Genes with two copies.

## Data Availability

The original contributions presented in this study are included in the article. Further inquiries can be directed to the corresponding authors.

## Supplementary Materials

The following supporting information can be downloaded at https://www.mdpi.com/article/10.3390/ijms27156935/s1.
