# Supplementary material for: The Pan-Plastome of Walnuts (Juglans regia L.) from Xinjiang Reveals New Insights into Their Genetic Diversity
Source: Int J Mol Sci. 2026 Aug 2;27(15):6935. doi: 10.3390/ijms27156935 (PMC13467509; doi:10.3390/ijms27156935)
Supplement: Supplementary file 1 [file ijms-27-06935-s001.zip › Supplementary Table S1.pdf]

Table S1. Information on 41 *Juglans regia L.* samples.

Sample ID	Accession	Sample origin	Longitude (°E)	Latitude (°N)
XJ2	Yecheng 2	Yecheng County, Kashgar Prefecture	77.31	36.93
XJ4	Yecheng 6	Yecheng County, Kashgar Prefecture	77.31	36.93
XJ5	Yecheng 7	Yecheng County, Kashgar Prefecture	77.31	36.93
XJ6	Yecheng 8	Yecheng County, Kashgar Prefecture	77.31	36.93
XJ7	Yecheng 9	Yecheng County, Kashgar Prefecture	77.31	36.93
XJ8	Yecheng 10	Yecheng County, Kashgar Prefecture	77.31	36.93
XJ10	Yecheng 15（12）	Yecheng County, Kashgar Prefecture	77.31	36.93
XJ12	20-15	Hotan County, Hotan Prefecture	79.30	36.23
XJ15	21-1	Hotan County, Hotan Prefecture	79.30	36.23
XJ19	22-5	Hotan County, Hotan Prefecture	79.30	36.23
XJ24	Xinxin 2	Xinhe County, Aksu Prefecture	81.88	41.34
XJ25	Wen 179	Wensu County, Aksu Prefecture	80.47	41.62
XJ26	Xinlu	Wensu County, Aksu Prefecture	80.47	41.62
XJ29	Xincuifeng	Wensu County, Aksu Prefecture	80.47	41.62
XJ30	Xinguang	Xinhe County, Aksu Prefecture	81.88	41.34
XJ31	Xinfeng	Hotan County, Hotan Prefecture	79.30	36.23
XJ36	Zha 346	Wensu County, Aksu Prefecture	80.47	41.62
XJ37	Zhaxin 14	Wensu County, Aksu Prefecture	80.47	41.62
XJ38	Zha 71	Wensu County, Aksu Prefecture	80.47	41.62
XJ39	Zha 200	Wensu County, Aksu Prefecture	80.47	41.62
XJ40	Zha 210	Wensu County, Aksu Prefecture	80.47	41.62
XJ41	Zhalureng	Wensu County, Aksu Prefecture	80.47	41.62
XJ42	Hechun06	Hotan County, Hotan Prefectureg	79.30	36.23
XJ43	Yi01	Aksu City, Aksu Prefecture	80.86	40.50
XJ44	Yexiu2	Yecheng County, Kashgar Prefecture	77.31	36.93

Table S1. Information on 41 *Juglans regia L.* samples.

Sample ID	Accession	Sample origin	Longitude (°E)	Latitude (°N)
XJ45	Sawa4	Yecheng County, Kashgar Prefecture	77.33	36.93
XJ46	Xinwu417	Wushi County, Aksu Prefecture	79.21	41.29
XJ47	Damuma	Hotan County, Hotan Prefecture	79.30	36.23
XJ48	Zha 343	Wensu County, Aksu Prefecture	80.47	41.62
BW1	BW1	Wensu County, Aksu Prefecture	80.47	41.62
CL1	CL1	Cele County, Hotan Prefecture	81.12	37.42
GL	Gongliuyehetaowang	Gongliu County, Ili Kazakh Autonomous Prefecture	81.73	43.86
H1	H1	Hotan County, Hotan Prefecture	79.30	36.23
JW1	JW1	Wensu County, Aksu Prefecture	80.47	41.62
M2-1	M2-1	Moyu County, Hotan Prefecture	79.98	38.14
PKK3	PKK3	Pishan County, Hotan Prefecture	78.49	37.13
QW3	QW3（North7-West3）	Wensu County, Aksu Prefecture	80.47	41.62
W1	W1	Wushi County, Aksu Prefecture	79.21	41.29
WAK1	WAK1	Wushi County, Aksu Prefecture	79.21	41.29
Wen185	Wen 185	Wensu County, Aksu Prefecture	80.47	41.62
JC	JC	Jiacha County, Xizang	92.72	29.27
